# The effect of sodium‐glucose cotransporter 2 inhibitors and glucagon‐like peptide 1 agonists on cardiovascular disease in patients with type 2 diabetes

**DOI:** 10.1002/clc.23152

**Published:** 2019-02-06

**Authors:** Amit K. Dey, Jacob Groenendyk, Nehal N. Mehta, Evgenia Gourgari

**Affiliations:** ^1^ Section of Inflammation and Cardiometabolic Disease, National Heart Lung and Blood Institute National Institutes of Health Bethesda Maryland; ^2^ Division of Pediatric Endocrinology Georgetown University Washington District of Columbia

**Keywords:** diabetes, SGLT‐2 inhibitors, GLP‐1 agonists

## Abstract

Patients with type 2 diabetes have a significantly increased risk of cardiovascular disease (CVD) compared to the general population—with CVD accounting for two out of every three deaths in patients with diabetes. In 2008, the FDA suggested that CVD risk should be evaluated for any new antidiabetic therapy, leading to a multitude of large CVD outcome trials to assess CVD risk from these medications. Interestingly, several of these outcome trials with new novel antidiabetic therapies have demonstrated a clear and definite CVD advantage at mid‐term follow up in high‐risk patients with T2DM. In this review, we discuss two relatively new classes of diabetic drugs, sodium‐glucose cotransporter 2 inhibitors and glucagon‐like peptide 1 agonists, and their efficacy in improving cardiovascular outcomes.

## 1 INTRODUCTION

Cardiovascular disease (CVD) is the leading cause of morbidity and mortality in patients with type 2 diabetes mellitus (T2DM), who have a 2 to 3‐fold increased risk of cardiovascular mortality compared to those without diabetes.^1^ However, significant progress has been made toward the mitigation of this increased CVD risk in diabetes. The all‐cause mortality rate of the Framingham study participants with diabetes has decreased by 48%, from 33.1 per 1000 person‐years in the early study period (1950‐1975) to 15.8 in more recent years (1976‐2001). An even stronger trend is present for cardiovascular mortality; a 69% decrease from 24.1 per 1000 person‐years in 1950 to 1975 to 6.8 in 1976 to 2001.^1^ Consistently, data from the NHANES study for the years 1971 to 2000 showed a similar trend in men, although a change in mortality rate among women with diabetes was not observed.^2^ Another large epidemiologic study analyzed trends in the incidence of diabetes‐related complication from 1990 to 2010 and found a large relative decline of 67.8% in acute myocardial infarction (95% confidence interval [CI], −76.2 to −59.3) among patients with diabetes.^3^

However, even with significant progress patients with diabetes remain at elevated risk of cardiovascular morbidity and mortality.^3^ Importantly, the excess mortality and comorbidity seen in patients with diabetes are more evident in patients who are younger, have poor glycemic control or have renal complications.^4^ Observational data have shown that an increase in glycated hemoglobin level of 1% corresponds to a 15% increased risk of incident CVD.^4^ There are some conflicting data in regards to cardiovascular outcomes from large studies that aimed to achieve excellent glycemic control. Data from the ACCORD trial showed increased mortality associated with intensive glucose control (glycated hemoglobin level below 6.0%), probably because of frequent hypoglycemic episodes associated with very strict glycemic control.^5^, ^6^ Lack of benefit in cardiovascular mortality from intensive glucose control has also been confirmed by other large, randomized controlled trials, such as the ADVANCE and the Veterans Affairs Diabetes Trial.^7^, ^8^ However, large trials, such as the UKPDS, have found reduction in myocardial infarction and death after 10 years of follow up with strict glucose control.^9^ Therefore, it appears there is a limit to the reduction of CVD burden that can be achieved by increasing the intensity of glucose control. While reaching glycated hemoglobin targets for all patients remains an important goal, other strategies for the prevention of CVD are needed. In this review, we discuss two relatively newer classes of medication for diabetes mellitus that have been increasingly recognized in recent years as agents that could assist in the prevention of CVD, namely SGLT‐2 inhibitors and glucagon‐like peptide 1 (GLP‐1) agonists.

### 1.1 SGLT‐2 inhibitors

The first nonspecific SGLT inhibitor to come into medical use was phlorizin, which is extracted from the bark of apple trees.^10^ Subsequently, it was discovered to cause glycosuria.^11^ However, therapeutic use of phlorizin in diabetes was limited because of low oral bioavailability.^12^ Investigators hypothesized that targeted inhibition of SGLT‐2, the sodium‐glucose cotransporter that resorbs 80% to 90% of the 180 g of sugar daily filtered by the kidney, could improve glycemic control in diabetes.^13^ Recently, specific inhibitors of SGLT‐2, such as dapagliflozin, canagliflozin, and empagliflozin have been developed for glycemic control in diabetes.^14^, ^15^, ^16^

Patients using SGLT‐2 inhibitors typically see a mild reduction in blood pressure, mild weight loss (approximately 2 kg loss at 1 year for empagliflozin, compared to 0.5 kg gain on placebo), and a small increase in both HDL and LDL cholesterol.^17^, ^18^, ^19^, ^20^ In addition, data from leptin‐receptor deficient mice suggests that treatment with SGLT‐2 inhibitors may decrease insulin resistance conferring additional benefits independent of glycemic control.^21^ The most common known side effect of these drugs is an increase in urinary tract infections, although concerns have also been raised about increased rates of diabetic ketoacidosis, bone fractures, and bladder cancers.^22^ Increased complaints of polyuria, associated with the osmotic diuresis phenomenon of SGLT‐2 inhibitors, have also been documented, along with concurrent hemoconcentration.^22^ Safety alerts have been issued by the US Food and Drug Administration (FDA) about the increased risk of decreased bone mineral density, leg or foot amputations (canagliflozin), and risk of ketoacidosis with SGLT‐2 inhibitors.^23^

In 2008, the US FDA suggested that new antidiabetic therapies be evaluated for associated alterations of cardiovascular risk, given concerns that specific glucose‐lowering drugs might worsen cardiovascular outcomes (CVOs).^24^, ^25^ Empagliflozin was the first novel SGLT‐2 inhibitor with results on cardiovascular outcomes. The EMPA‐REG OUTCOME trial (Table 1), which randomized patients to receive 10 mg empagliflozin, 25 mg empagliflozin, or placebo once daily, was a multi‐center trial that included 7020 adults at high cardiovascular risk (based on history of cardiovascular disease) and with glycated hemoglobin between 7.0% and 9.0% (or 10.0% if on stable glucose‐lowering therapy).^26^ The primary outcome of EMPA‐REG OUTCOME, which was a composite of cardiovascular death, non‐fatal myocardial infarction, and non‐fatal stroke, was significantly less likely to occur in the pooled empagliflozin group (10.5%) compared to the placebo group (12.1%, *P* = 0.04). The secondary outcome, cardiovascular death, occurred in 12.8% of patients in the empagliflozin group compared to 14.3% of the placebo group, although this difference was not statistically significant (*P* = 0.08).^26^ Interestingly, there was not a significant change in the rate of myocardial infarction or stroke between the two groups; hospital admission for heart failure, however, was significantly less likely in the empagliflozin group (occurring in 2.7%) vs the placebo group (4.1%, *P* = 0.002).^26^ After the first 12 weeks of the trial, during which glucose‐lowering therapy was held stable, those in the empagliflozin group had glycated hemoglobin levels about 0.5 percentage points lower than those in the control group, with little difference between the 10 and 25 mg/day groups.^26^ At 4 years, the difference between the empagliflozin groups and the placebo groups was about 0.3%, although adjustment of antiglucose medications was allowed after week 12.^26^ Rates of serious adverse events, adverse events, and overall rates of urinary tract infection and pyelonephritis were similar between the empagliflozin groups and the placebo group, although adverse, severe adverse and serious adverse events, genital infections, and urinary tract infections were significantly more in the pooled empagliflozin group compared to the placebo group.^26^

**Table 1:** Characterization of all trials

Trial	CANVAS	DECLARE TIMI	EMPA REG OUTCOME	ELIXA	LEADER	SUSTAIN‐6	EXSCEL	HARMONY
Intervention	Canagliflozin or placebo	Dapagliflozin or placebo	10 or 25 mg empagliflozin vs placebo	Lixisenatide or placebo	Liraglutide (median 1.78 mg daily) or placebo	0.5 or 1.0 mg of semaglutide once weekly vs placebo	2 mg exenatide vs placebo	30‐50 mg s.c albiglutide vs placebo
Number of patients	10 142	17 160	7020	6068	9340	3297	14 752	9463
Inclusion criteria (CV risk)	65.6% hx of cv disease. Either >30 years and hx of ASCVD or > 50 years with multiple risk factors	40 years of age or older and had type 2 diabetes, a glycated hemoglobin level of at least 6.5% but less than 12.0%, and a creatinine clearance of 60 mL or more per minute.	History of cardiovascular disease	MI or hosp. For unstable angina in previous 180 days	Age > 50 years with at least one coexisting cardiovascular condition OR age >60 years with at least one CV risk factor	Age > 50 and previous cardiovascular, cerebrovascular, or peripheral vascular disease, NYHA class II or III, or stage 3 CKD or age > 60 with CV risk factors	Any level of CV risk	Men and women aged 40 years or older with a diagnosis of type 2 diabetes and established disease of the coronary, cerebrovascular, or peripheral arterial circulation who had a glycated hemoglobin concentration of more than 7·0% (53 mmol per mole) were eligible for participation in the trial
Primary outcome	CV death, nonfatal MI, nonfatal stroke	MACE, and a composite of cardiovascular death or hospitalization for heart failure	CV death, nonfatal MI, nonfatal stroke	CV death, MI, stroke, hosp. For unstable angina	CV death, nonfatal MI (including silent), nonfatal stroke	CV death, nonfatal MI, nonfatal stroke	CV death, nonfatal MI, nonfatal stroke	Composite outcome, which comprised death from cardiovascular causes, myocardial infarction, and stroke
Baseline glycated hemoglobin	8.20%	8.30%	8.10%	8.10%	8.70%	8.70%	8.00%	8.76%
Median follow‐up	126 weeks	4.2 years	3.1 years	25 months	3.8 years	2.1 years	3.2 years	1.6 years
MACE	26.9 vs 31.5 (placebo) events/1000 person‐years	8.8% in the dapagliflozin group and 9.4% in the placebo group; hazard ratio, 0.93; 95% CI, 0.84 to 1.03; *P* = 0.17	37.4 vs 42.9 (placebo) events/1000 person‐years (*P* = 0.04)	13.4% vs 13.2% (placebo) *P* = 0.81	13.0% vs 14.9% in placebo, HR 0.87, *P* = 0.01	3.2 vs 4.4 (placebo) events/100 person‐years , *P* = 0.02	3.7 vs 4.0 (placebo) events/100 person‐years	4·57 events per 100 person‐years in the albiglutide group and at 5·87 events per 100 person‐years in the placebo group (HR 0·78, 95% CI 0·68‐0·90), *P* = 0·0006
All‐cause mortality	17.3 vs 19.5 (placebo) per 1000/person‐years	6.2% in the dapagliflozin group and 6.6% in the plcacebo group; hazard ratio, 0.93; 95% CI, 0.82 to 1.04	19.4 vs 28.6 (placebo) per 1000 person‐years, *P* < 0.001	3.1 vs 3.3 (placebo) per 100 person‐years , *P* = 0.50	8.2% (2.1 per 100 person‐years ) vs 9.6% (2.5 per 100 person‐years ), *P* = 0.02	1.8 vs 1.8 per 100 person‐years , *P* = 0.79	2.0 vs 2.3 (placebo) per 100 person‐years	0·95 (95% CI 0·79‐1·16) compared to placebo

Abbreviations: CI, confidence interval; CV, cardio vascular; HR, hazard ratio; MACE, Major Adverse Cardiovascular Events; MI, myocardial infarction.

Subsequently, the effects of treatment with canagliflozin, another SGLT2 inhibitor on cardiovascular events were investigated in the CANVAS and CANVAS‐R studies, which were published in combined form as the CANVAS Program (Table 1).^27^ The combined trials included 10 142 patients in 30 countries, with a minimum follow‐up of 78 weeks (median 126 weeks).^27^ Notably, while all CANVAS Program patients were at high cardiovascular risk based on the presence of risk factors, only 65.6% had history of cardiovascular disease, compared to >99% in the EMPA‐REG OUTCOME trial.^27^ Similar to the EMPA‐REG OUTCOME study, participants treated with canagliflozin saw an average decrease of 0.6% in glycated hemoglobin and about 1.6 kg in body weight when compared to patients receiving placebo at follow‐up.^27^ The event rate for the primary outcome, a composite of cardiovascular death, non‐fatal myocardial infarction, or non‐fatal stroke, was observed significantly less in patients randomized to canagliflozin than those randomized to placebo (drug vs placebo: events in 26.9 vs 31.5 participants per 1000 patient‐years, hazard ratio (HR) 0.86, *P* = 0.02).^27^ Event rates for the secondary outcome (death from any cause) did not statistically differ, at 17.3 vs 19.5 events per 1000 patient‐years (*P* = 0.24).^27^ Serious adverse events were more likely to occur in the placebo group, at 120.0 adverse events per 1000 patient‐years, compared to the canagliflozin group, at 104.3 adverse events per 1000 patient‐years (*P* = 0.04).^27^ However, the canagliflozin group experienced a greater rate of amputation (6.3 vs 3.4 events per 1000 patient‐years, *P* < 0.001); infection of male genitalia (34.9 vs 10.8, *P* < 0.001); mycotic genital infection in females (68.8 vs 17.5, *P* <0.001); bone fractures (15.4 vs 11.9, *P* = 0.02); and volume depletion (26.0 vs 18.5, *P* = 0.009).^27^

More recently, the effects of treatment with dapagliflozin, another SGLT2 inhibitor upon cardiovascular events were evaluated in the DECLARE—TIMI trial, which randomized 17 160 patients with type 2 diabetes and either established cardiovascular disease or multiple cardiovascular risk factors to 10 mg dapagliflozin or placebo once daily.^28^, ^29^ Participants treated with dapagliflozin did not result in a lower rate of major adverse cardiovascular events (MACE) (8.8% in the dapagliflozin group and 9.4% in the placebo group; HR, 0.93; 95% CI, 0.84‐1.03; *P* = 0.17) but resulted in a lower rate of cardiovascular death or hospitalization for heart failure (4.9% vs 5.8%; HR, 0.83; 95% CI, 0.73‐0.95; *P* = 0.005) when compared with placebo.^30^ A meta‐analysis of 9339 patients enrolled in either phase 2b (5 studies) or phase 3 (16 studies) trials of dapagliflozin found a non‐significant trend towards benefit in event rates of MACE (1.15 per 100 patient‐years in dapagliflozin groups vs 1.69 per 100 patient‐years, HR 0.77 95% CI 0.54‐1.10).^31^ Of note, dosages ranged from 2.5 to 10 mg dapagliflozin daily and some studies included a comparator group rather than a placebo. Smaller randomized trials have shown similar change in body weight and blood pressure at 24 weeks to those observed with other SGLT‐2 inhibitors.^32^

In addition, Wu et al performed a meta‐analysis of six regulatory submissions (37 525 participants) and 57 published trials (33 385 participants), which included seven different SGLT‐2 inhibitors.^33^ The authors found that the relative risk (RR) of cardiovascular death was 0.63 (0.51‐0.77, *P* < 0.0001) in favor of those treated with SGLT‐2 inhibitors and the RR of MACE was 0.84 (0.75‐0.95, *P* = 0.006).^33^ Non‐fatal stroke risk, with RR 1.3, was borderline increased (1.00‐1.68, *P* = 0.049). Notably, over 50% of the participants included in this meta‐analysis were from the EMPA‐REG OUTCOME study.^33^ A recent meta‐analysis that included 82 SGLT‐2 trials and 1968 major cardiovascular events further confirmed that SGLT2 inhibitors were protective against major cardiovascular events, heart failure, as well as all‐cause mortality.^34^ When interpreting the effects of SGLT‐2 inhibitors on cardiovascular outcomes it is important to consider the beneficial effects of concurrent antihypertensive therapies on these outcomes. The new 2017 ACC/AHA guidelines recommend a treatment goal of less than 130/80 mm Hg for patients with diabetes.^35^ Although these studies were performed prior to the new hypertension guidelines in 2017,^36^ most patients were on some degree of blood pressure control therapy. Approximately, 80% of patients in both the EMPA‐REG OUTCOME and CANVAS Program studies were on a renin‐angiotensin‐aldosterone system modifying medication at baseline, with 65% and 54% on a beta blocker and 43% and 44% on a diuretic in each trial, respectively. Baseline systolic blood pressures were below the 2014 guidelines set by the eighth Joint National Committee (less than 140/90 mm Hg for those under 60 years, less than 150/90 mm Hg for those older than 60 years).^26^, ^27^, ^37^ Once the new, lower goals for blood pressure treatment have been fully adopted, it will be important to reassess the effectiveness of the glucose‐modifying medications described in this systematic review. Future investigation will be needed to elicit whether these beneficial effects of SGLT‐2 inhibitors are still present in patients treated under the 2017 ACC/AHA guidelines.^35^

The mechanism through which SGLT2 inhibitors affect changes in cardiovascular outcomes is not entirely known. It is suspected that, in addition to the decrease in glucose levels, small but favorable effects on other CV risk factors contribute. These include decrease in body weight, favorable modifications to lipid levels, and lower blood pressure.^29^ It is unlikely that inhibition of the SGLT2 pathway has direct effects on cardiac tissue; while SGLT1 receptors have been demonstrated to be present in the heart, SGLT‐2 receptors have not.^29^ The precise mechanisms of the CV effects of SGLT2 inhibitors still remain to be fully understood. Ultimately, more mechanistic studies with longer follow‐up are needed to understand the nature of CV effects and determine whether these cardioprotective effects are sustained.

### 1.2 GLP‐1 agonists

In 1964, Elrick et al showed that oral glucose administration stimulated greater insulin secretion than intravenous glucose administration.^38^ These effects were later showed to be driven by gut‐derived incretins, such as glucagon‐like peptide 1 (GLP‐1). GLP‐1 is a peptide secreted from the gut in response to food intake. GLP‐1 can improve glucose control in patients with diabetes through several mechanisms which include enhanced insulin secretion from beta cells, delayed gastric emptying, and inhibition of glucagon secretion. Furthermore, GLP‐1 induces satiety which can lead to weight loss and subsequently improved insulin resistance. These observations have led to the development of a new class of drugs that enhance the action of GLP‐1, GLP‐1 agonists.^39^, ^40^ Postulated mechanisms of action of this new class of drug include increase in insulin levels, decrease in glucagon levels as well as delay in gastric emptying.^41^, ^42^ Native incretin hormone GLP‐1 has a very short plasma half‐life and thus its antihyperglycemic effects can only be exploited by augmenting its half‐life. This can be achieved by exendin‐based therapies (exenatide, exenatide once weekly), dipeptidyl peptidase‐4‐resistant analogs (lixisenatide, albiglutide), and analogues of human GLP‐1 (liraglutide, taspoglutide) which in turn have different half‐lives.^43^

The LEADER trial, one of the first GLP‐1 agonist trials evaluated the effects of the addition of liraglutide, a GLP‐1 agonist, on MACE, defined as CV death, nonfatal MI, or nonfatal stroke, in 9340 patients with diabetes and elevated cardiovascular risk when compared with placebo.^44^ 72% of these patients had established cardiovascular disease at baseline; mean glycated hemoglobin level was 8.7%.^44^ After a median of 3.8 years of follow up, 13.0% of patients in the liraglutide group had experienced MACE, compared to 14.9% in the placebo group (*P* = 0.01, HR 0.87).^44^ While the overall rate of myocardial infarction was slightly lower in the liraglutide group (1.6 vs 1.9 per 100 patient‐years in placebo, *P* = 0.046), no significant trends were observed in rates of fatal MI, stroke, or hospitalization for heart failure^.^ 44 At 36 months, patients in the liraglutide group experienced an average decrease in weight of 2.3 kg more than those in the placebo group, in addition to a 1.2 mm Hg decrease in systolic blood pressure and an increase in heart rate of 3.0 beats per minute. Patients in the liraglutide group had elevated amylase and lipase compared to those in the placebo group, as well as a non‐significantly elevated incidence of pancreatic carcinomas (0.3% vs 0.1%, *P* = 0.06).^44^ Glycated hemoglobin levels in the liraglutide group were 0.40 percentage points lower than those of the placebo group^.^ 44 The FIGHT trial evaluated the use of liraglutide in patients recently hospitalized with heart failure and reduced left ventricular ejection fraction and did not show any clinical benefit post heart failure hospitalization.^45^ The LIVE trial evaluated the use of liraglutide on patients with advanced cardiomyopathy and chronic heart failure. This study not only confirmed prior results but also showed that liraglutide is associated with an increase in heart rate as well as more serious cardiac adverse events in patients with chronic heart failure and reduced left ventricular function.^46^

Another independent GLP‐1 agonist study, the ELIXA study, evaluated the effects of lixisenatide when compared to placebo in 6068 diabetes patients who had a history of myocardial infarction or hospitalization for unstable angina in the previous 180 days^.^ 47 The primary outcome was a composite of cardiovascular death, myocardial infarction, stroke, or hospitalization for unstable angina^.^ 47 This end point occurred in 13.4% of patients in the lixisenatide group and 13.2% of patients in the placebo group (*P* = 0.81 for superiority); 4.0% of patients in the lixisenatide group and 4.2% in the placebo group were hospitalized for heart failure (*P* = 0.75).^47^ Average glycated hemoglobin level difference across all visits was 0.27 percentage points lower for those in the lixisenatide group compared to the placebo group^.^ 47

Furthermore, the SUSTAIN‐6 trial evaluated the effects on incidence of composite cardiovascular death, nonfatal stroke, and nonfatal MI of 0.5 mg or 1.0 mg of another GLP‐1‐agonist, semaglutide, given once weekly against placebo in 3297 patients.^48^ Seventy‐two percent of patients had established CV disease at baseline, and mean glycated hemoglobin level was 8.7%. The primary composite outcome occurred in 6.6% of patients in the semaglutide group, compared to 8.9% in the placebo group (*P* = 0.02).^48^ Rates of all‐cause mortality and cardiovascular mortality were similar between both intervention and placebo groups; the rate of nonfatal stroke (0.80 per 100 person‐years) was significantly lower than the placebo group (1.31/100 person‐years , *P* = 0.04).^48^ The mean glycated hemoglobin level, compared to the placebo group, decreased by 0.7 percentage points in patients in the 0.5 mg semaglutide group and 1.0 percentage points in the 1.0 mg semaglutide group (*P* < 0.001 for both).^48^ Mean body weight at week 104 was 2.9 kg lower in the 0.5 mg group and 4.3 kg in the 1.0 mg group, compared to placebo; systolic blood pressure was reduced by 3.4 and 5.4 mm Hg vs placebo, respectively^.^ 48 Treatment discontinuation in the semaglutide group was higher than that in the placebo group, largely driven by significantly increased incidence of nausea and vomiting in the semaglutide group.^48^

Finally, the EXSCEL study tested the effects of 2 mg exenatide, another GLP‐1 agonist, administered once weekly against placebo on the incidence of cardiovascular death, nonfatal MI, or nonfatal stroke in 14 752 patients followed for a median period of 3.2 years.^49^ 73% of enrolled patients had previous cardiovascular disease, and baseline glycated hemoglobin level was 8.0%.^49^ Mean glycated hemoglobin level was 0.7 percentage points lower in the exenatide group at 6 months when compared to the placebo group (*P* < 0.001), though this difference did narrow over time.^49^ The observed event rates for the primary outcome were 3.7 events/100 person‐years for the exenatide group, compared to 4.0 events/100 person‐years in the placebo group (*P* = 0.06).^49^ There was not a significant difference in the incidence rate of serious adverse events between the two groups.^49^ Even though overall mortality was lower in the exenatide treatment group, the investigators noted that the threshold for significance was not met with this outcome according to the pre‐specified hierarchical analysis plan^.^ 49 The mechanisms for improved CV outcomes with semaglutide and liraglutide but not with lixisenatide remain to be elucidated. At this point, it is not clear whether the improved CVOs are due to a class‐effect of GLP‐1 agonists or due to specific medication properties only, but future clinical trial will help answer this question. Previous animal studies and clinical trials suggest possible CV benefits of GLP‐1 agonists through modulation of CV risk factors including weight loss, reduction in blood pressure, improved lipid metabolism, and effects on the vascular endothelium.^.^ 50

This year, the HARMONY trial investigated almost 9500 patients treated with either albiglutide (which was removed from the market in 2017) or placebo for a median 1.6‐year follow‐up period. Albiglutide was shown to significantly reduce the risk of MACE by 22%; however, the study did not show a significant reduction in death from cardiovascular causes, as was demonstrated with liraglutide, another GLP‐1 agonist in the LEADER trial.^51^ This finding may be related to the short duration of follow‐up in the study.

The ongoing REWIND trial is a randomized placebo‐controlled trial which will test the cardiovascular effects of once‐weekly dulaglutide, another GLP‐1 agonist in patients with type 2 diabetes. The highlight of the trial is its high proportion of women and a predominantly primary prevention population.^52^Another ongoing trial PIONEER 6 is designed as a non‐inferiority trial that will investigate the cardiovascular safety of oral semaglutide, another GLP‐1 agonist compared with placebo.^53^ Both these trials will provide important insight for regulatory approval of GLP‐1 agonists.

Collectively, the overall signal that seems to emerge from the literature about the effects of these novel drugs is a positive effect on CV outcomes among patients at risk for structural heart disease, a lack of effects on heart failure outcomes among those with early cardiac remodeling, and possible detrimental effects on heart failure outcomes in patients with advanced symptomatic heart failure.^54^, ^55^ The new ADA standards along with the EASD guidelines, recently recommended the use of SGLT2 inhibitors in patients with cardiovascular disease and diabetes.^56^ Moreover, ACC consensus committee published a statement endorsing the use of GLP‐1 agonists along with metformin in patients with T2DM with established atherosclerotic cardiovascular disease after having a thorough physician‐patient discussion regarding risk‐benefit.^57^ Finally, although these novel therapies have demonstrated a clear and definite cardiovascular disease advantage at mid‐term follow up, results from longer term follow up are awaited. Considering these trials have been short‐mid‐term follow‐up studies, prospective studies are needed to investigate the effects on long‐term vascular endpoints and mortality.

## 2 CONCLUSIONS

In summary, SGLT‐2 inhibitors (empagliflozing and canagliflozin) and GLP‐1 agonists (liraglutide, semaglutide) have been shown to improve CVOs in patients with T2DM. The American Diabetes Association 2018 standard of care guidelines recommended for the first time the addition of a second antihyperglycemic agent that can reduce MACE in select patients with T2DM and pre‐existing atherosclerotic cardiovascular disease.^30^ Future clinical trials will investigate whether these beneficial effects are sustained over time and whether other agents can have similar results. It is certainly important that new options for the prevention of CVD in patients with T2DM have become available, that go beyond the traditional treatments with hypertensive and lipid‐lowering medications. Whether the extended use of these drugs will eventually decrease the CVD mortality in the large population of patients with T2DM remains to be examined in future epidemiologic studies.

## CONFLICTS OF INTEREST

The authors declare no potential conflicts of interests.
